# A Laminar Flow-Based Microfluidic Tesla Pump via Lithography Enabled 3D Printing

**DOI:** 10.3390/s16111970

**Published:** 2016-11-23

**Authors:** Mohammed-Baker Habhab, Tania Ismail, Joe Fujiou Lo

**Affiliations:** Department of Mechanical Engineering, Bioengineering Program, University of Michigan at Dearborn, 2088 IAVS Building, 4901 Evergreen Rd., Dearborn, MI 48128, USA; mihabhab@umich.edu (M.-B.H.); tismail@umich.edu (T.I.)

**Keywords:** Tesla turbine, Tesla valve, microfluidic, DLP, 3D printing

## Abstract

Tesla turbine and its applications in power generation and fluid flow were demonstrated by Nicholas Tesla in 1913. However, its real-world implementations were limited by the difficulty to maintain laminar flow between rotor disks, transient efficiencies during rotor acceleration, and the lack of other applications that fully utilize the continuous flow outputs. All of the aforementioned limits of Tesla turbines can be addressed by scaling to the microfluidic flow regime. Demonstrated here is a microscale Tesla pump designed and fabricated using a Digital Light Processing (DLP) based 3D printer with 43 µm lateral and 30 µm thickness resolutions. The miniaturized pump is characterized by low Reynolds number of 1000 and a flow rate of up to 12.6 mL/min at 1200 rpm, unloaded. It is capable of driving a mixer network to generate microfluidic gradient. The continuous, laminar flow from Tesla turbines is well-suited to the needs of flow-sensitive microfluidics, where the integrated pump will enable numerous compact lab-on-a-chip applications.

## 1. Introduction

**Figure 1:**
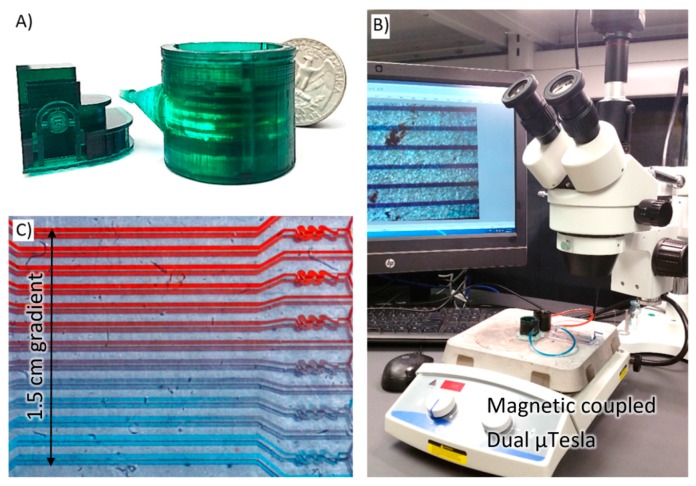
3D printed µTesla pump. (**A**) The assembled pump is around an US quarter in diameter, printed in high resolution as demonstrated by the miniature engineering building of UM Dearborn; (**B**) Two µTesla pumps were coupled magnetically to the same stir plate to ensure exact pressure delivered to a microfluidic device, and visualized under a stereomicroscope; (**C**) Using this setup, gradients can be generated by µTesla pump to drive a microfluidic mixer network.

In the early 1900s, Nikola Tesla, a Serbian inventor, introduced a new turbine design that replaced the conventional impeller blades with smooth disks [1,2]. This design places multiple disks in parallel on a rotating shaft. Around the shaft are holes that allow fluids to flow orthogonal to the disks. Fluid is injected tangential to the disks and spirals across the disc surface towards the orthogonal holes at the center. Due to the viscous effects on the boundary surfaces of the parallel disks, the fluid then transfers its momentum towards the rotating disks continuously. As the discs begin to rotate, the path of the fluid begins to increase in length, which in turn increases the momentum transfer. In his 1913 patent for the “Tesla turbine”, Tesla originally anticipated this design to be used for geothermal power generation with an efficiency over 80% [3,4,5]. However, the turbine can also be operated in reverse where rotation can be applied to drive a smooth tangential flow from the parallel disks. For example, pumping with Tesla turbine which is pulseless in nature has been demonstrated in a number of applications including the artificial heart [6,7]. Furthermore, the transfer of momentum is dependent on the boundary layer at the walls of the parallel disks, which can be improved by laminar flows within the low Reynold number regime. Thus the Tesla turbine is well suited to microfluidic flow. To achieve a miniature microfluidic Tesla (µTelsa) pump with complex disk and shaft geometries, a Digital Light Processing (DLP) based 3D printer was employed here (Projet 1200, 3DSystems, Rock Hill, SC, USA), enabling prototypes to be built with 43 µm × 43 µm × 30 µm voxel resolution. A multidisc rotor was completely enclosed in a 3D printed housing and magnetically coupled to a simple laboratory stir plate, reducing the complexity of mechanical drive and eliminating the need for fluidic sealing, Figure 1. Using this setup, the pump pressure, flow, and power output were characterized at multiple fluidic loads and visualized under a stereo microscope. Lastly, the pump was connected to a microfluidic mixer network with special Tesla valve geometries, demonstrating an all-Tesla-fluidics system for lab-on-a-chip applications.

### 1.1. Microfluidic Pumps

It can be asserted that a complete lab-on-a-chip device requires a source of fluidic power. Towards this, many efforts have been devoted to miniaturize pumps or create a driving force for flow in a microchannel. Typical external flow sources rely on screw-type syringe pumps, rotor-type peristaltic pumps, or press-driven pumps to control flow in the µL to nL per minute domain. Novel integrated microfluidic pumps include accoustofluidic pumps [8], the technology used in inkjet print heads [9]; electroosmotic pumps [10], which apply voltage across a diffusion membrane to control ionic flow; electrolysis gas pressure pumps [11]; capillary force siphoning [12], à la paper microfluidics [13,14]; and a number of valve-based peristaltic pumps [15,16] such as those applied in commercial miniaturized insulin pumps (STMicroelectronics, Santa Clara, NV, USA) [17]. Additionally, a number of aforementioned pumps, especially peristaltic ones, utilize passive and asymmetrical flow valves [18,19]. These special valves, which often coincidentally apply Tesla’s other work in fluidics, the Tesla valve [20], in order to achieve low fluidic resistance in one flow direction, and much higher resistance in the opposite. However, most of the aforementioned microfluidic pumps cannot provide smooth laminar flow due to a pulsatile driving force like valves and acoustics, while others required special voltage that energized ionic membranes and produced flat-top electroosmotic flows. The benefit of a miniaturized Tesla pump leverages the fluidic mechanics of laminar flow within parallel disks, which is well-suited and specifically designed to match the low Reynolds number of the microfluidic channels that it is intended to drive. 

### 1.2. Theoretical Design of the µTesla Pump

The design of the turbine geometry in the µTesla pump was proceeded in the manner similar to previously published designs of the Tesla turbine [21,22,23,24]. First, a cylindrical coordinate system was adapted to describe the spiraling flow path between the parallel turbine disks. Then, the flow conditions including Reynold number, etc. were defined. Next, the gap distance was derived from the flow conditions. And finally, the number of disks was specified in accordance with the flow rate requirements. Details of this design process is described in the following.

First, the governing equation for steady incompressible laminar flow between adjacent disks were cylindrically described by:

(1) $$\frac{1}{r}\frac{\partial \left(rV_{r}\right)}{\partial r}+\frac{1}{r}\frac{\partial V_{\theta }}{\partial \theta }+\frac{\partial V_{z}}{\partial z}=0$$

V denotes the velocity components where r,θ are the radius and angle of the cylindrical coordinate system. The following assumptions were taken [25]: (1) the flow is two-dimensional and constant across the channel between the rotor plate surfaces and are mean velocities; (2) the flow field and velocity components are constant across the gaps, with body force represented by the wall shear at each (r,θ) location; (3) the flow field is radially symmetric and the inlet flow at rotor outer edge is uniform and θ derivatives of flow are therefore zero; (4) radial pressure gradients are negligible compared to angular momentum and frictional forces the wall. These assumptions reduce the continuity Equation (1) to:

(2) $$\frac{1}{r}\frac{\partial \left(rV_{r}\right)}{\partial r}=0$$

From the Navier-Stokes equation, the *r*-direction and θ-direction momentums reduce to:

(3) $$V_{r}\frac{\partial V_{r}}{\partial r}-\frac{{V_{\theta }}^{2}}{r}=-\frac{1}{\rho }\left(\frac{\partial P}{\partial r}\right)+f_{r}$$

(4) $$V_{r}\frac{\partial V_{r}}{\partial r}+\frac{V_{r}V_{\theta }}{r}=f_{\theta }$$

while the z-direction moment and pressure gradient are both zero. 

Next, the following flow conditions were considered throughout the turbine design: In order to maintain laminar flows between the rotor disks, Reynolds number was designed at 1000, below the typically values associated with transition into the turbulent regime. The spin rate of the rotor ω was nominally set at 500 rpm (stir plate maximum of 1200 rpm). And the volumetric flow rate Q was designed to be 70 µL/min, an appropriate range for the microfluidic flows the pump is designed to drive. Thus, to calculate the gap distance, we start with the mass flow rate between the disk gaps:

(5) $$VA\rho =2\pi rbV_{r}\rho =\frac{{\dot{m}}_{c}}{n}$$

where ${\dot{m}}_{c}$ is the total mass flow rate, b is the gap size between rotating plates, 2πrb is the circumferential disk gap area, and n is the number of disk gaps.

The gap thickness is related to the hydraulic diameter by:

(6) $$D_{h}=2b$$

and the Reynolds number *Re*, designed at 1000, is a function with hydraulic diameter, viscosity, and velocity of the fluid, here described by:

(7) $$Re=\frac{\rho \Delta V_{\theta }D_{h}}{\mu }=\frac{\Delta V_{\theta }D_{h}}{\nu }$$

where $\Delta V_{\theta }$ is taken to be the tangential velocity at the disk edge, $D_{h}$ is the hydraulic length, and ν is the kinetic viscosity, $D_{h}$ can be specified at 1.65 mm giving a gap b of 0.826 mm.

Finally, the total volumetric flow rate is divided into individual flows between each pair of disks. And thus the total flow rate is described by:

(8) $$N=n+1,\;\mathrm{where}\;n=\dot{Q}/q$$

where $\dot{Q}$ is the designed fluid flow rate, i.e., 70 µL/min, q is the flow rate between each pair of disks, n is the number of disk gaps, and N is thus the total number of disks.

On the other hand, the total mass flow rate ${\dot{m}}_{c}$ is equal to the density times volumetric flow rate. Applying this to Equation (5) gives:

(9) $$V_{r}2\pi r_{0}b\rho =\frac{{\dot{m}}_{c}}{n}\;=\frac{\rho \cdot \dot{Q}}{n}=\rho \cdot q,\;\mathrm{thus}\;q=V_{r}2\pi r_{0}b$$

**Figure 2:**
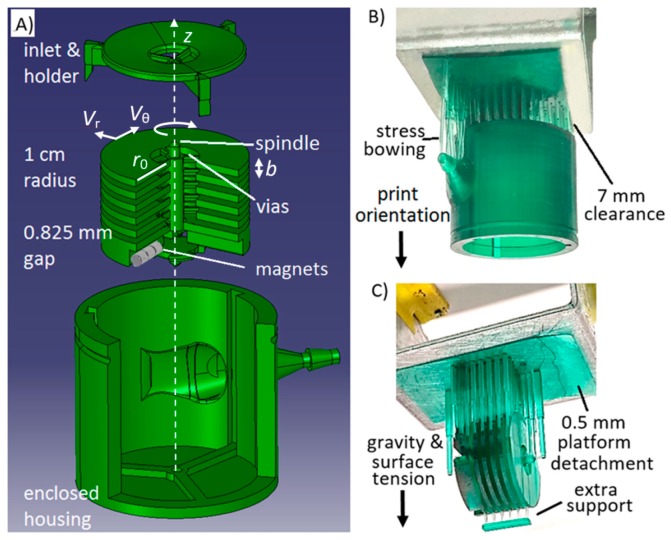
Computer design and 3D printing of µTesla. (**A**) The µTesla pump was comprised of three parts: cap with pivot, the rotor with vias and pockets for magnets, and the enclosed housing with fluidic spout and bottom pivot. The computer design was then printed in a DLP 3D printer. The print was upside down as pictured in (**B**,**C**) and subjected to gravity pull; (**B**) To reduce surface tension pulling down large areas of the enclosed housing’s bottom, substantial clearance was required along with numerous supporting posts to prevent excessive warping and detachment; (**C**) The rotor required extra support to keep the disks straight. Additional resin platform was added to ensure adhesion to the metal print stage.

$V_{r}$ is taken to be the radial velocity of the fluid being pumped out of the disks, which is equal to the total design flow rate $\dot{Q}$ divided by the cross sectional area of the gaps between the disks [26]. With the designed rotation speed, turbine disk radius of 1 cm, and b defined at 0.826 mm, $V_{r}$ was 2.57 × 10^−6^ m/s, q was 1.32 × 10^−10^ m^3^/s, and *n* was ~5, giving us six disks. The µTesla design parameters are summarized in Table 1 and applied to the CAD design for 3D printing, Figure 2A.

## 2. Rapid Prototyping Using DLP 3D Printer

Lithography based 3D printing requires the object to be built layer by layer from a bath of photo curable resin. This technique has a marvelous appearance as the object is literally “pulled out” of the resin bath. 3D printing has been used in the rapid prototyping of self-enclosed microfluidics [27,28], stereolithography-based microfluidics [29,30,31], miniaturized electronics [32], and microoptical systems [33,34,35], representing a *key* enabling technology to achieve intricate overhanging three-dimensional structures in microfabrication. This key technology was leveraged to shrink the complex µTesla geometries—with multiple disks, ports, vias—to a microscale dimension matching the microfluidic flow for which it is designed to drive.

### Addressing DLP Printing Issues

The µTesla pump was printed in three parts—cap holder, rotor, and nozzled housing—and assembled to complete the device, Figure 2A. To translate the µTesla design into a 3D build, we must consider the DLP lithography issues in the following details. First, the minimum feature size differs from the actual DLP resolution (pixel pitch) or print thickness. The minimum wall thickness, for example, was found to be 0.5 mm to prevent collapse during printing. The minimum overhang and enclosed cross-sectional diameter was designed to be 1.5 mm, e.g., the nozzle lumen, as smaller dimensions would trap resin and the structure will not be resolved. However, supported, non-overhanging structures can be routinely achieved with 70–100 µm minimum feature sizes. Secondly, as each layer of the 3D build is exposed and pulled away from the print surface, the liquid-solid interface repeatedly exerts surface tension on the build structure. Hence adequate support structures for large lateral surfaces is required to counter this surface tension during printing. For example, the large base of the pump housing required numerous support beams with minimum of 7 mm clearance from the print stage, Figure 2B. If inadequate support leads to detachment or warpage, the resultant protruding material tends to splash unwanted print resin randomly, creating print defects. Thirdly, another technique to counteract surface tension was to include a bulk resin adhesion layer, which prevented detachment of supports near the edges of large structures, Figure 2C. In all final builds, this 0.5 mm thick support resin platform was included. Lastly, the lithographically printed structures often exhibited residual stress after the post-curing step. This was found to be partially reduced if the isopropanol washing step before post-curing was limited to two 60-s rinses, to limit solvent swelling of the polymer. Nevertheless, noticeable amount of residual stress remained, as seen in the bowed support beams, and were addressed by additional lateral support structures, Figure 2C.

## 3. Results of µTesla Performance

The µTesla was mounted to a standard stir plate with speed control graduated from 0 to 1200 rpm as shown in Figure 1. The µTesla rotor speed is coupled to the stir plate’s rotation, which we have also verified via slow motion videos, Supplementary Video S1. Using this setup, the performances of the µTesla was quantified as follows.

### 3.1. µTesla Hydraulic Head and Stall Characteristics

**Figure 3:**
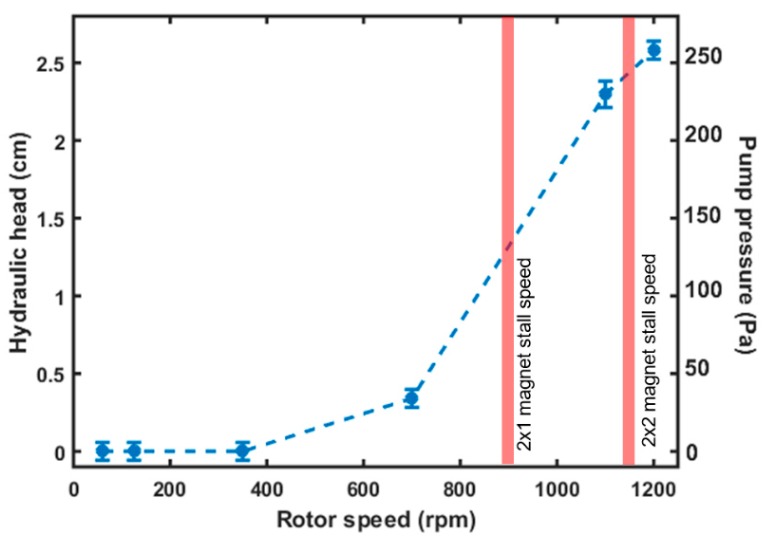
Hydraulic head. The height above the µTesla pump to which the fluid was raised was characterized for rotor speeds from 60 to 1200 rpm (a 15 cm, 1/16 ID Tygon tubing was coupled for this measurement). With a pair of single magnet to couple the stir plate rotation, the rotor stalled around 900 rpm. With a pair of double magnets, stalling occurred around 1150 rpm. The total hydraulic head at 1200 rpm corresponds to 253 Pa of pressure. Error bar denotes standard deviation from 6 measurements.

The hydraulic pressure developed by the µTesla was characterized by measuring the height of the water column that was pumped up a connected Tygon tube with 1/16” inner diameter. Tygon tubes at varying lengths were also used for subsequent flow characterizations. First, the µTesla was primed with isopropanol to reduce the bubbles trapped between the rotor disks and tubing. Then, water was injected using a 5 mL syringe from the Tygon tubes. During the characterization, it was found that the pump rotor would stall at specific speeds if there was inadequate holding force from the magnets. Therefore, single, double, and triple 0.4 lb. magnets (2 mm ∅ × 1.5 mm, McMaster-Carr, Aurora, OH, USA) were placed at opposite ends of the rotor to test the stall characteristic when the µTesla was primed with water. The results showed that two sets of 3 magnets coupled end to end were required to eliminate stalling and provide speeds up to 1200 rpm, technically limited by our stirring plate, Figure 3. Furthermore, at 1200 rpm, the maximum hydraulic head achieved was 2.6 cm, equivalent to 253 Pa of developed pressure. In comparison, a microfluidic bladed turbine required almost 4000 rpm to produce 200 Pa of pressure [36].

### 3.2. Flow Characteristics of µTesla at Different Resistances

**Figure 4:**
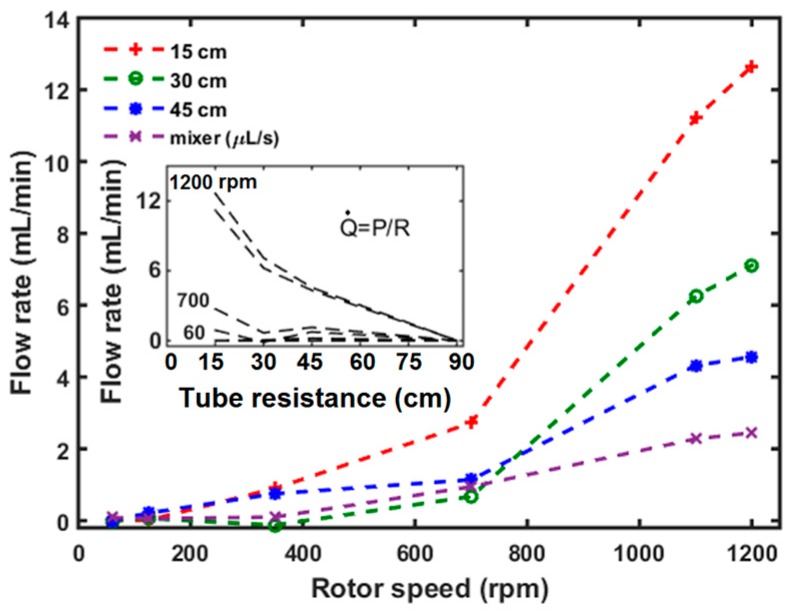
Flow output at different loads. The flow characteristics of the µTesla pump under load was measured with different lengths of Tygon tubing (1/16″ ID), and through a microfluidic mixer device. The flow rate across the mixer device is considerably lower at 0–14.6 µL/min, and plotted in a different unit. Inset: Flow and fluidic resistance is inversely proportional. By extrapolating the microfluidic flow rates, its resistance is equivalent to a tubing length of 90 cm, or 1.04 MPa·s/m^3^.

Next, the µTesla pump was loaded with increasing fluidic resistances created by varying lengths of Tygon tubing from 15 to 45 cm, and connected to a microfluidic mixer (described in Section 4). The outlet tubing or microfluidic was placed at the same level as the pump spout to eliminate pressure differences due to height. The output volume from the tubing or microfluidics was then collected directly into a microcentrifuge tube for 30 s. This volume was then quantified via manual micro pipetting and calculated for the flow rate per minute. From Figure 4, it can be seen that the flow output increased with rotor speed, but began to taper close to 1200 rpm. The maximum flow output at 1200 rpm achieved with the 15 cm tubing was 12.6 mL/min. The flow across the mixer microfluidic achieved a significantly lower rate of 0–14.6 µL/min owing to its increased fluidic resistance. At a fixed rotor speed, flow output and resistance were inversely proportional, as expected from the constant pressure generated by the pump. Extrapolating the data from the 15–45 cm tubing, the mixer device has a fluidic resistance equivalent to 90 cm of tube length, with 1.04 MPa·s/m^3^. This is comparable to the higher end of fluidic resistances typically found in microchannels [37].

### 3.3. µTesla Pump Power Calculations

**Figure 5:**
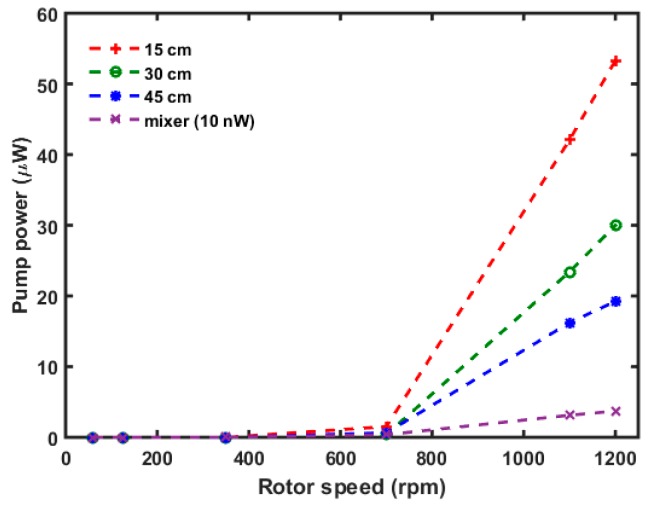
Pump power at different loads. The hydraulic power can be calculated from the product of hydraulic head and flow rates. Power drops after different fluidic loads were also calculated, including load across a microfluidic mixer. The µTesla produced 53 µW of power at 1200 rpm across the lowest load of 15 cm. The power reduced to 37 nW across the mixer microfluidic load. The 53 µW of µTesla’s nominal power output was more than adequate to drive the mixer device at flow rates of 0–14.6 µL/min, as shown in Figure 4.

Given the characteristic pressures and flow rates that were measured, the hydraulic power output of the µTesla pump was calculated using Power = $\dot{Q}$·ρ·g·∆h (where $\dot{Q}$ is flow in m^3^/s and ρ·g·∆h is from the hydraulic head) (Figure 5). The maximum power output of µTesla at 15 cm resistance (nearly unloaded) was 53 µW. The power after the mixer load was dropped to 37 nW, which meant that most of the output power was consumed by pumping the mixer device at 1200 rpm. Nevertheless, this power was capable of driving 0–14 µL/min of flow in the mixer device, adequate for general microfluidic applications. 

### 3.4. Laminar Flow Velocity Profile in Microchannels

The µTesla pump was connected to a microfluidic mixer and velocity profile inside the channel was quantified with nanoparticle velocimetry using dark field microscopy. 70 nm spherical gold nanoparticles were diluted in water and pipetted inside the microchannel before connecting the µTesla pump.

**Figure 6:**
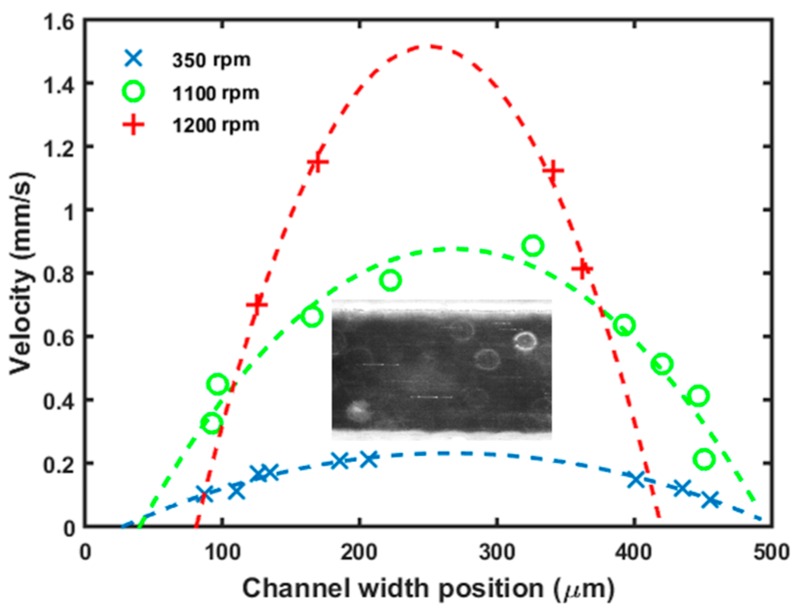
Flow profile at different pump rates. To demonstrate that the pump is able to create laminar flow in the microfluidic mixer, a 500 µm wide section of a microchannel was imaged with gold nanoparticles in the flow stream (lines in insert). With the camera integration times fixed at 100–200 ms, the streak length was calibrated and calculated to yield the flow rate across the width of the channel. Parabolic flow profiles were observed at the three rotor speeds tested.

Flow across a channel of 500 µm by 500 µm cross section was then visualized under dark field to observe the nanoparticle scattering. The majority of the nanoparticles aggregated to form bright clusters easily visible even under short integration times. The pictures taken at 100 and 200 ms integration times, tailored to the speed of the flow, were analyzed in ImageJ. The calibrated distances of the particle streaks, Figure 6 inset, was divided by the integration time to yield particle velocity. As expected at the flow rates under 14 µL/min, velocity profile mimics the parabolic shapes in the laminar regime.

## 4. Results of Driving the Tesla Inspired Microfluidic Mixer

**Figure 7:**
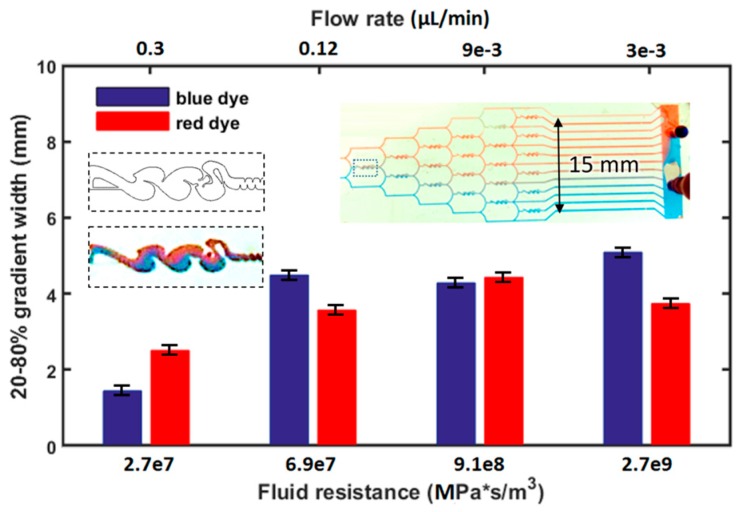
µTesla driven, Tesla-inspired mixer microfluidics. The all-Tesla-fluidic system generated gradients using blue and red dyes at flow rates as low as 3 nL/min. The mixer incorporates flow folding structures similar to Tesla valves (CAD and enlarged insert on left). The widest gradient achieved spanned 4 mm across (20%–80% intensity), as analyzed from their RGB channels in ImageJ. Errors bars denote maximum and minimum values from image analysis.

To demonstrate the usage, two µTesla pumps were coupled on the same laboratory stir plate, Figure 1, to guarantee the same flow rates that pumped red and blue food dye into the microfluidic mixer network (Figure 7 insert). This particular network incorporates mixer geometries inspired by the Tesla-valve, another fluidic work that Nicholas Tesla patented in 1920 [20]. The valves were typically used to create asymmetric flow resistance, where flow in one direction has much higher resistance than another. Here, it is used to create folding flows to provide convective mixing inside the gradient generator network. Instead of tuning the pump speed, additional fluidic resistance in Tygon tubing was added to the microfluidics to reduce the flow rates. The resultant color mixing was captured under the stereomicroscope and analyzed by ImageJ. The blue and red channels were analyzed to find their 20% to 80% intensities, and the width between these were plotted against the fluidic resistances applied. As expected, we observed higher mixing at higher resistance, or low flow rates, Figure 7. The maximum gradient width achieved at 3 nL/min flow rate was 4 mm wide. This gradient was a demonstration of an all-Tesla-fluidics based system for microfluidic applications.

## 5. Future Works

Further miniaturization and integration into a single microfluidic substrate can open the doors to a number of µTesla based lab-on-a-chip applications. In order to achieve this, three future improvements should be made: (1) The print resolution can be improved, either through higher print resolution or lower surface tension platforms used in competing DLP systems; (2) the fluid path along the axial holes and the spout apertures needs to be optimized to account for differential pressure drops across the disks [38]; (3) electromagnetic integration would eliminate the stir plate and provide a self-powered device that can be visualized under both upright and inverted microscopes. Future studies on the temporal responses of the µTesla’s flow, both through simulation and flow visualization between the disks (a challenging task at the microscale level) can also reveal additional performance and efficiency characteristics of the µTesla pump. These works represent many exciting research directions this current work may lead to in the future.

## 6. Conclusions

A miniaturized Tesla turbine fluidic pump was designed and fabricated using DLP based 3D printing. The turbine geometries were designed with low Reynolds number of 1000. The DLP lithography 3D printing required the optimization of feature sizes, support structures, and gap to platform distances to minimize surface tension and residual stress that may warp the printed parts. The resultant µTesla pumps were coupled magnetically to a lab stirring plate to provide rotor actuation up to 1200 rpm, where it achieved a pump pressure of 253 Pa and maximum flow greater than 12 mL/min. Coupling this µTesla pump to a microchannel mixer with Tesla-valve inspired geometries generated gradients using color dyes. In this manner, a complete pump-and-microfluidic system was realized based on Tesla’s fluidic theories. Future miniaturization by optimizing the print resolution and integrating electronics has the potential to create a low cost, low complexity, self-powered, unified lab-on-a-chip device for bioassay applications.

## Figures and Tables

**Table 1:** µTesla Design Parameters.

**Reynold Number**	$Re_{x}$ = 1000
**Rotational speed**	500 rpm→0.5236 m/s
**Disk radius**	$r_{0}=1\;\mathrm{cm}$
**Volumetric flow rate**	Q=40 μL/min
**Disk Gap**	b = 0.826 mm, as D_h_ = 1.65 mm
**Number of disks**	N=6

## Supplementary Materials

The supplementary materials are available online at http://www.mdpi.com/1424-8220/16/11/1970/s1.
